# Supplementary material for: Targeted treatment of injured nestmates with antimicrobial compounds in an ant society
Source: Nat Commun. 2023 Dec 29;14:8446. doi: 10.1038/s41467-023-43885-w (PMC10756881; doi:10.1038/s41467-023-43885-w)
Supplement: Supplementary file 1 — Supplementary Information [file 41467_2023_43885_MOESM1_ESM.pdf]

Supplementary Information for

Antimicrobial woundcare of infected injuries in an ant society

Erik. T. Frank*, Lucie Kesner, Joanito Liberti, Quentin Helleu, Adria C. LeBoeuf, Andrei Dascalu, Douglas B. Sponsler, Fumika Azuma, Evan P. Economo, Patrice Waridel, Philipp Engel, Thomas Schmitt, Laurent Keller

Correspondence to: erik.frank@uni-wuerzburg.de

This PDF file includes:

Supplementary Figures 1 to 8
Supplementary Tables 1 to 13

Other Supplementary Files for this manuscript include the following:

Supplementary Movies 1 to 2
Supplementary Data 1

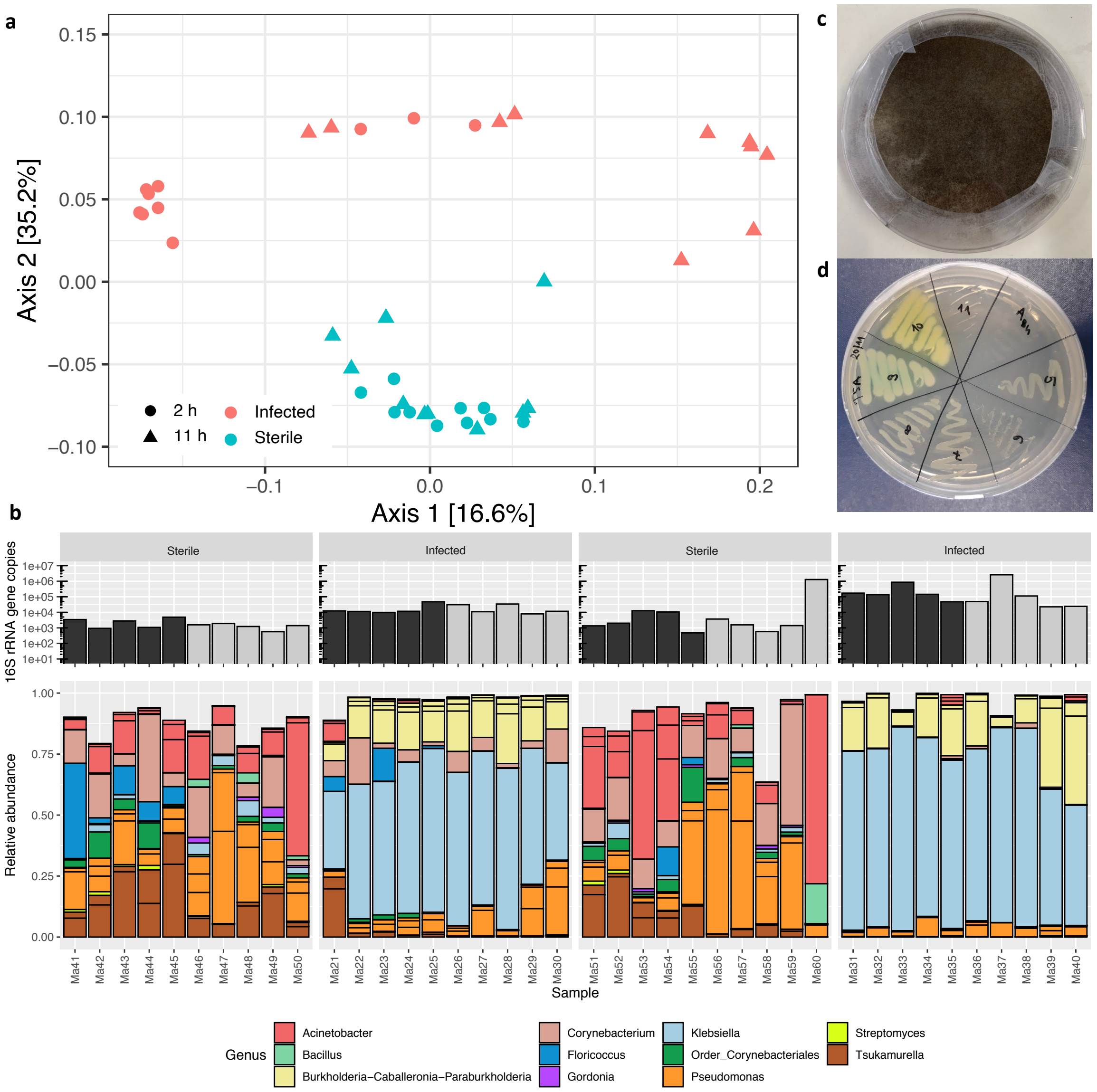

Supplementary Fig. 1 | Ant microbiome and soil pathogens. (a) Principal Coordinate Analysis of the microbiome of the thorax of sterile (blue) and infected ants (red) at 2 and 11 hours (as seen in Fig. 1b). ADONIS: Treatment: $F_{(1,39)}$=17.45; R^2=0.31; P<0.001. N=10 for all groups. **(b)** 16S rRNA gene copy numbers and relative abundance of bacterial genera present in the thorax of the same individuals as in Fig. 1A. Multiple bars of the same color indicate different amplicon-sequence variants (ASVs) belonging to the same genera. ADONIS: Treatment: $F_{(1,39)}$=17.45; R^2=0.31; P<0.001. N=10 for all groups. **(c)** Microbial culture of surface soil grown on an agar plate (mostly showing fungal mycelia of *Rhizopus* with black spores). **(d)** Bacterial cultures of isolated *Pseudomonas aeruginosa* strains from the soil. Cultures in panels c and d were repeated 10 times showing the same result.

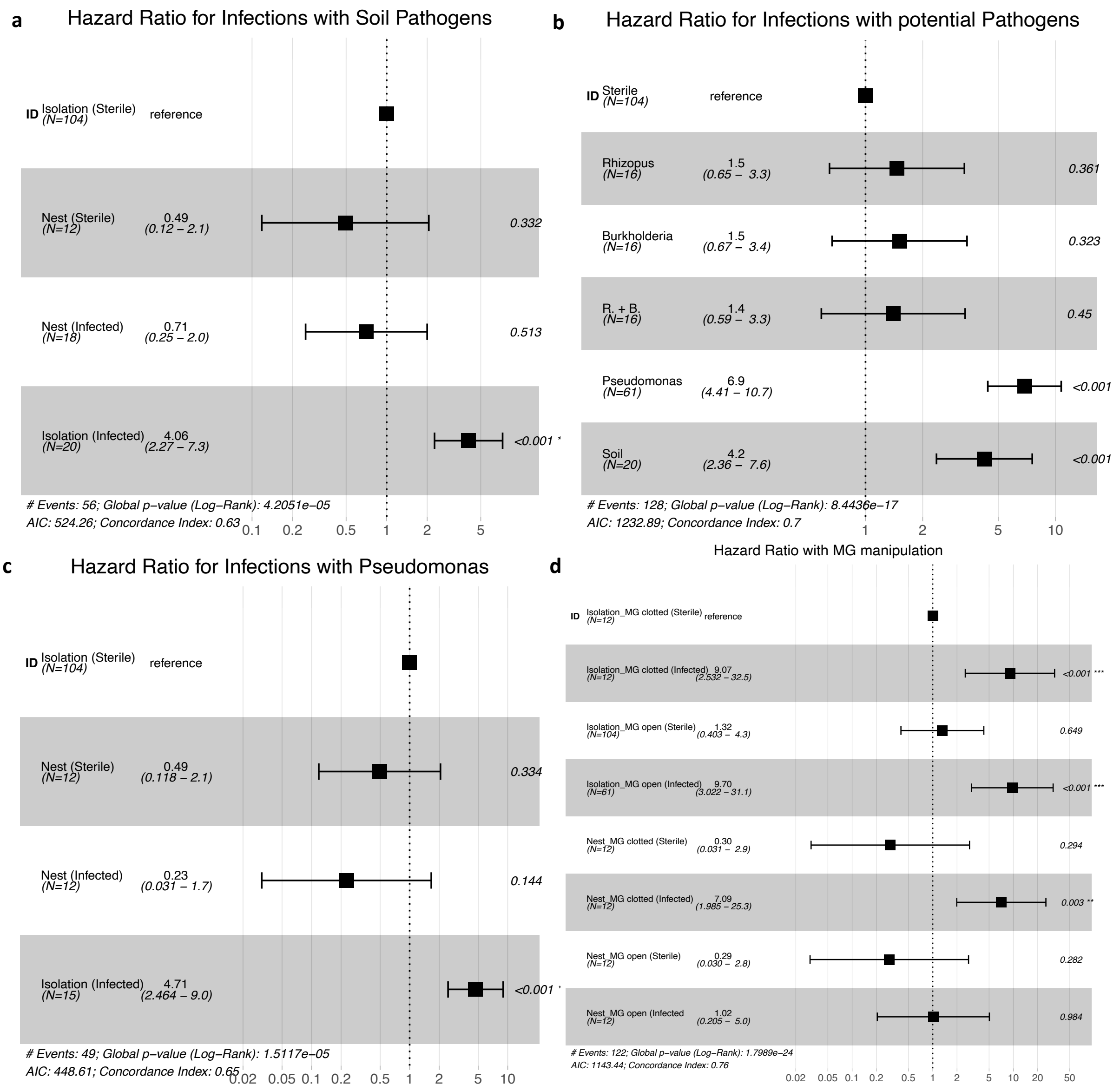

Supplementary Fig. 2 | Cox proportional hazard models for survival of infections. The reference were sterile ants in isolation with colony of origin as random factor. **(a)** Statistical results for infected ants (surface soil pathogens: OD=0.1) in isolation and inside the nest (Fig. 1c). **(b)** Statistical results for infected ants kept in isolation exposed to different potential pathogens cultured from the soil (*Burkholderia*, *Rhizopus*, *Pseudomonas*; OD=0.1) or surface soil pathogens (Supplementary Fig. 3). **(c)** Statistical results for infected and sterile ants (*P. aeruginosa*: OD=0.05) in isolation and with nestmates in the nest (Fig. 2a). **(d)** Statistical results of infected ants (*P. aeruginosa*: OD=0.05) kept in isolation or inside sub-colonies containing workers with or without plugged MG openings (Fig. 4; Supplementary Fig. 5).

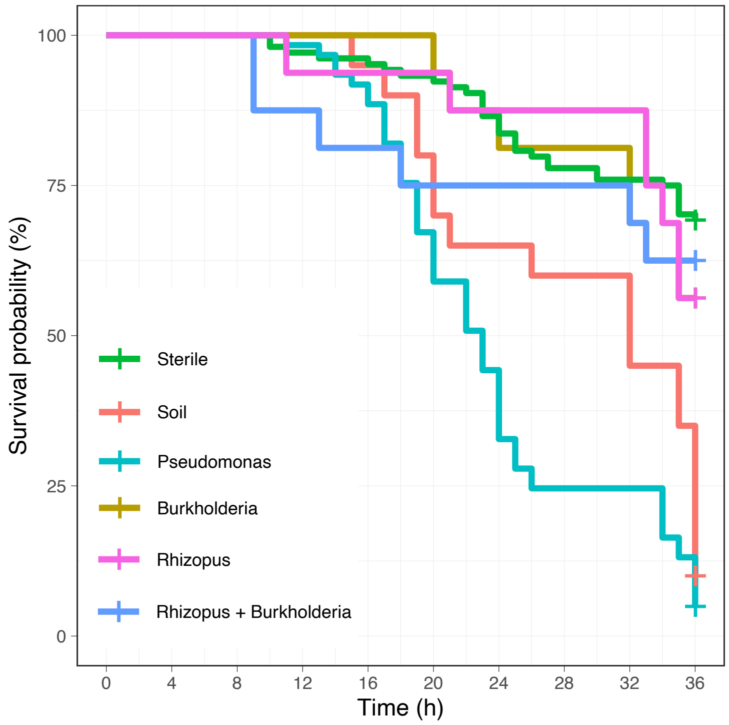

Supplementary Fig. 3 | Survival probability for different pathogen types. Kaplan – Meier cumulative survival rates of workers kept in isolation chambers. All individuals had one hind leg cut off at the femur, the wound was then exposed to a sterile PBS solution (green, sterile, *n*=104), a mix of either surface soil pathogens (red, Soil OD=0.5, *n*=20) or isolated pathogens diluted in PBS (OD=0.1): *P. aeruginosa* (blue, *Pseudomonas*, *n*=61), *Burkholderia* (brown, *n*=16), *Rhizopus* (pink, *n*=16), or *Rhizopus* with *Burkholderia* (dark blue, *n*=16). The treated ants were observed for 36 hours and the time of death noted. Statistical significance tested with a mixed-effects Cox proportional hazards regression model (Supplementary Fig. 2b).

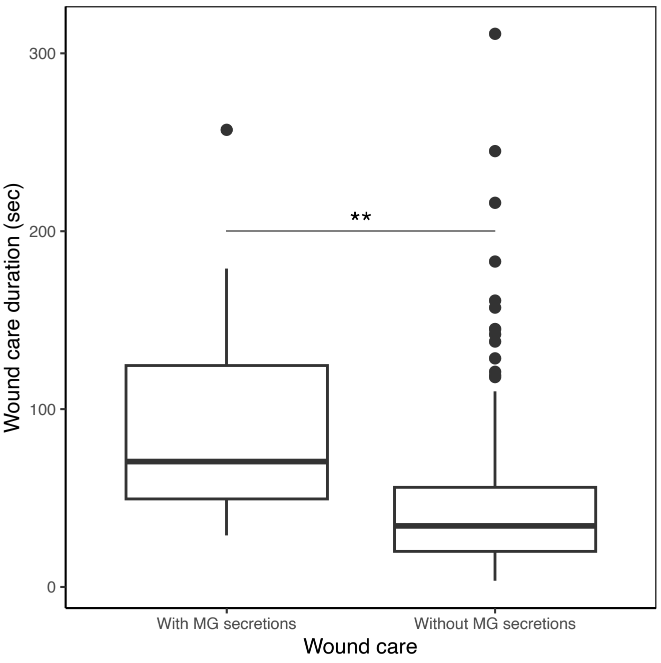

Supplementary Fig. 4 | Differences in wound care duration with and without metapleural gland secretions. Duration of wound care with and without MG secretions for the first 24 hours after injury inside the nest. With metapleuralgland (MG) secretions: wound care involving the MG for both infected and sterile ants pooled together using either the MG of the injured ant (*n*=26 for infected, *n*=10 for sterile ants) or of the nursing ant (*n*=2 for infected, *n*=4 for sterile ants), total sample size *n*=42. Without MG secretions: wound care excluding the MG for both infected and sterile ants pooled together (*n*=174 for infected, *n*=192 for sterile ants), total sample size *n*=366. Linear mixed effect model (Random Factor: Individual: Variance=134.4, Std. Dev.=11.59; Residual: Variance=2671.2, Std. Dev.=51.68) with two-sided Satterthwaite's t-test for wound care duration with and without MG secretions: DF= 47.92; t=-3.09; *P*=0.003**.

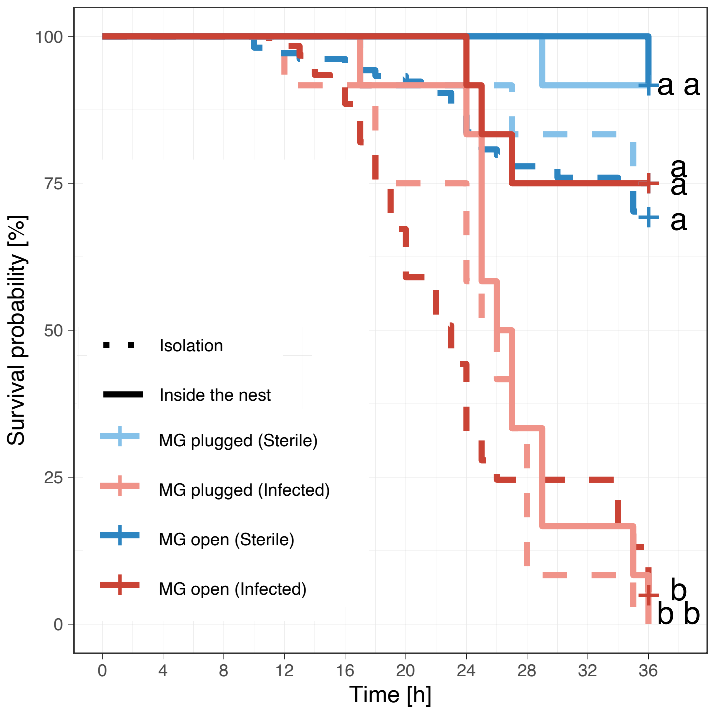

Supplementary Fig. 5 | Importance of MG secretions on survival of sterile and infected ants. Kaplan – Meier cumulative survival rates of workers in isolation (dotted line) or inside sub-colonies (solid line) whose wounds were exposed to *P. aeruginosa* diluted in PBS (red, Infected, OD=0.05) or a sterile PBS solution (blue, Sterile). In three sub-colonies all ants had their metapleuralgland (MG) opening closed with acrylic color (lighter color shade, MG plugged), while it remained open in the other three sub-colonies (darker color shade, MG open). *n*=12 per treatment. Detailed statistical results in Supplementary Fig. 2d and Supplementary Table 5, significant differences were calculated using a two-sided least square means with Holm-Bonferroni correction.

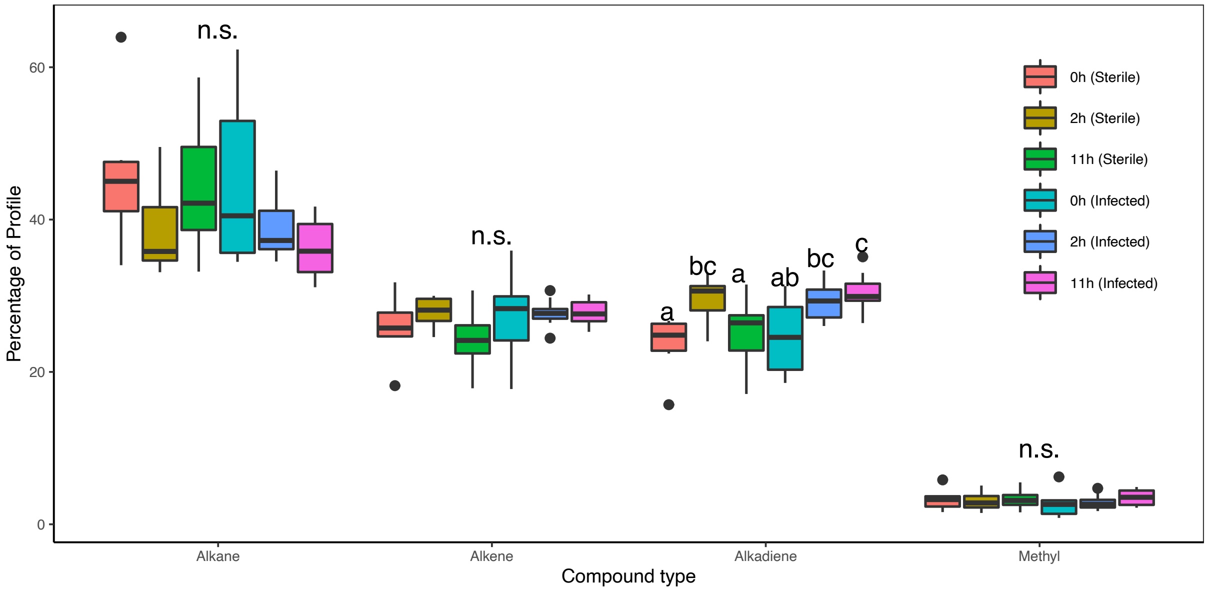

Supplementary Fig. 6 | Chemical composition of cuticular hydrocarbon profiles of sterile and infected ants. Differences across compound types (alkanes, alkenes, alkadienes and methyl-branched alkanes) for sterile ants (Sterile; *n*=30) and ants infected with *P. aeruginosa* diluted in PBS (OD=0.05, Infected; *n*=30) at three different time points: 0h (n=6 sterile, n=6 infected), 2h (n=12 sterile, n=12 infected) and 11h (n=12 sterile, n=12 infected). Detailed statistical results in Supplementary Table 8. Significant differences ($P<0.05$) are shown with different letters and were calculated using a two-sided Tukey Honest Significant differences test with Holm-Bonferroni correction. Boxplots show median (horizontal line), interquartile range (box), distance from upper and lower quartiles times 1.5 inter-quartile range (whiskers), outliers (>1.5x upper or lower quartile). Red: sterile ants after 0h, brown: sterile ants after 2h, green: sterile ants after 11h, light blue: infected ants after 0h, dark blue: infected ants after 2h, pink: infected ants after 11h.

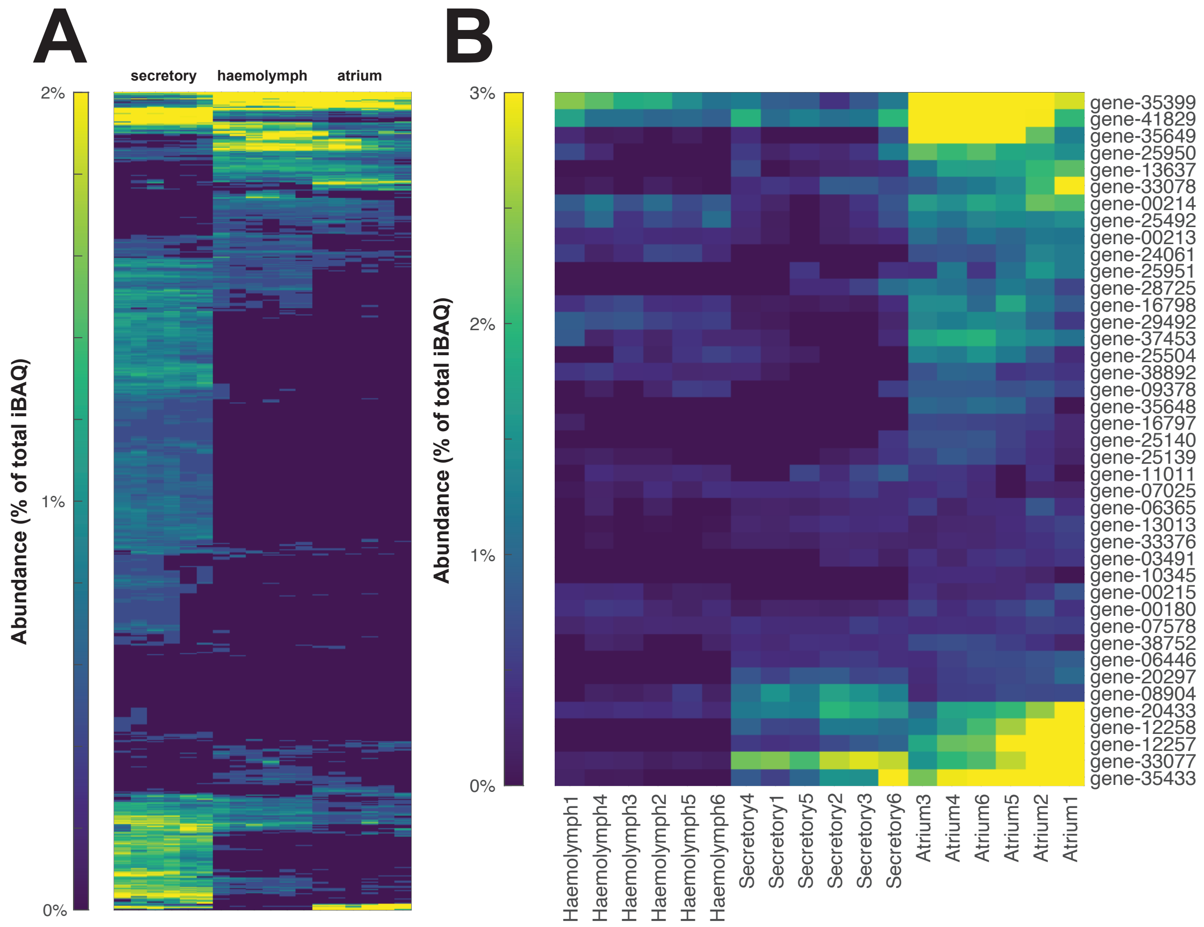

Supplementary Fig. 7 | Proteins from the metapleural gland. (a) All proteins identified in hemolymph (*n*=6), secretory cells (*n*=6) and metapleural gland atria (*n*=6). **(b)** The 41 proteins deduced through filtering to be secreted by the metapleural gland. Gene annotations can be found in Supplementary Table 12. Heatmap colors indicate normalized percentages of total iBAQ (intensity based absolute quantification) for a given sample.

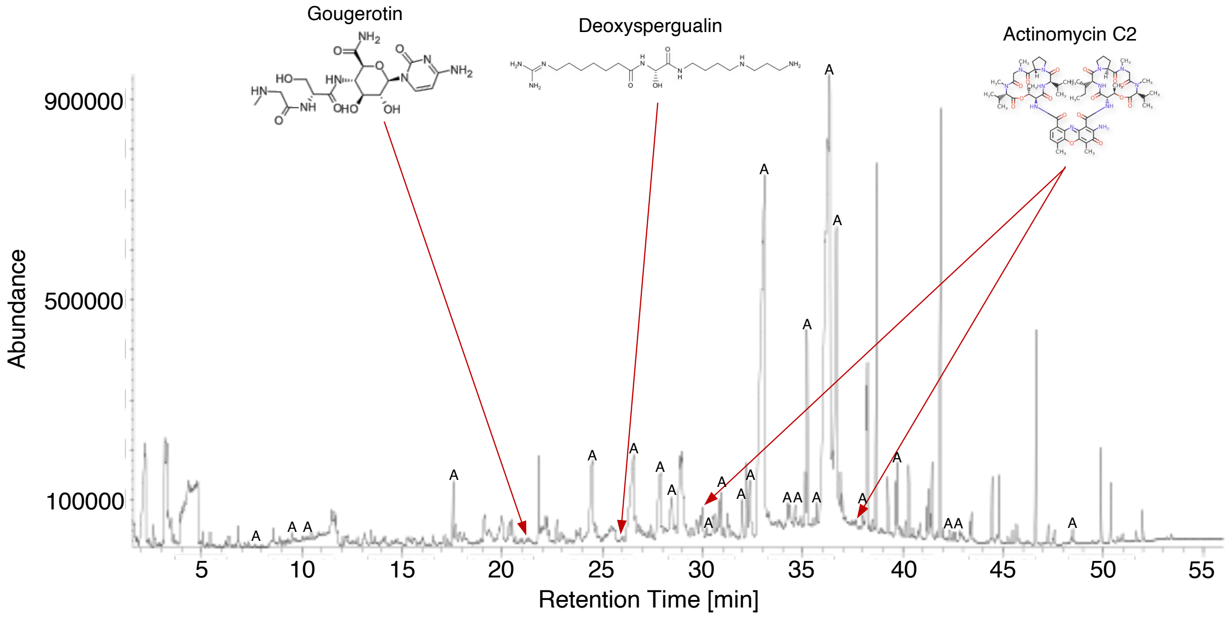

Supplementary Fig. 8 | Chemical compounds in the metapleural gland. Gas-chromatographic representation of one sample of 6 pooled metapleural glands (MG). Red arrows indicate chemical compounds with similar structure to known antibiotics and antimicrobials. Peaks marked with the letter A represent carboxylic acids. A detailed list of all chemical compounds found in the MG is described in Supplementary Table 12.

**State 1**	**State 2**	***t ratio***	***P***
2h – Sterile	11h – Sterile	-1.54	0.26
2h – Sterile	2h – Infected	-3.08	0.012*
2h – Sterile	11h – Infected	-6.75	<0.001***
11h – Sterile	2h – Infected	1.54	0.26
11h – Sterile	11h – Infected	-5.21	<0.001***
2h – Infected	11h – Infected	-3.66	0.003**

Supplementary Table 1 | Statistical differences in bacterial load (16S gene copies) between the treatments presented in Fig 1a. Linear mixed effect model (Random Factor: Colony: Variance=0, Std. Dev.=0; Residual: Variance=2.26, Std. Dev.=1.50; Likelihood ratio test of model vs intercept only model: X^2_3= 34.9, P<0.001), n=10 per group. Post-hoc analysis with least square means with Holm-Bonferroni correction for the 4 groups.

Permutation t-test results at 2 hours

Pathogen	R^2	*t*	*P*
Acinetobacter	0.15	-1.76	0.09
Bacillus	0.09	-1.32	0.2
Burkholderia	0.97	23.89	<0.001***
Corynebacterium	0.43	3.68	0.002**
Floricoccus	0.01	-0.48	0.6
Gordonia	0.01	-0.37	0.7
Klebsiella	0.92	14.73	<0.001***
Corynebacteriales	0.02	0.62	0.5
Pseudomonas	0.35	3.11	0.005**
Streptomyces	0.56	-4.79	<0.001***
Tsukamurella	0.03	-0.78	0.4

Permutation t-test results at 11 hours

Pathogen	R^2	*t*	*P*
Acinetobacter	0.07	-1.18	0.26
Bacillus	0.08	-1.26	0.2
Burkholderia	0.98	27.61	<0.001***
Corynebacterium	0.02	0.60	0.56
Floricoccus	0.01	-0.40	0.7
Gordonia	0.27	-2.58	0.02*
Klebsiella	0.81	8.65	<0.001***
Corynebacteriales	<0.01	-0.03	0.97
Pseudomonas	0.28	2.62	0.02*
Streptomyces	0.19	-2.04	0.08
Tsukamurella	0.01	0.31	0.8

Supplementary Table 2 | Statistical differences between infected and sterile ants in pathogen abundance for the different bacterial genera at 2 and 11 hours presented in Fig. 1b. Statistical results calculated with a permutation t-test with Holm-Bonferroni correction.

Mixed effects Cox Model

Experiment	Exp(coef)	*Z*	*P*
Nest – Sterile	0.47	-1.04	0.3
Nest – Infected	0.83	-1.32	0.75
Isolation – Infected	4.58	4.25	<0.001***

Least square means (post-hoc analysis)

Experiment 1	Experiment 2	*Z*	*P*
Isolation – Sterile	Nest – Sterile	1.040	0.89
Isolation – Sterile	Nest – Infected	0.324	1
Isolation – Sterile	Isolation – Infected	-4.246	<0.001***
Nest – Sterile	Nest – Infected	-0.630	1
Nest – Sterile	Isolation – Infected	-2.916	0.017*
Nest – Infected	Isolation – Infected	-2.759	0.023*

Supplementary Table 3 | Statistical differences for mortality between the different treatments presented in Fig 1c. Mixed effects Cox proportional hazards regression model: Random factor: Colony: Variance: 0.11, Std. Dev. 0.33. Likelihood ratio test of model vs intercept only model: $X^2_3= 16.4$, $P<0.001$. Post-hoc analysis with least square means with Holm-Bonferroni correction for the 4 groups.

Mixed effects Cox Model			
**Experiment**	**Exp(coef)**	***Z***	***P***
Nest – Sterile	0.43	-1.14	0.25
Nest – Infected	0.20	-1.59	0.11
Isolation – Infected	4.22	4.20	<0.001***

Least square means (post-hoc analysis)			
**Experiment 1**	**Experiment 2**	***Z***	***P***
Isolation – Sterile	Nest – Sterile	1.145	0.50
Isolation – Sterile	Nest – Infected	1.590	0.34
Isolation – Sterile	Isolation – Infected	-4.197	<0.001***
Nest – Sterile	Nest – Infected	0.638	0.52
Nest – Sterile	Isolation – Infected	-2.994	0.014*
Nest – Infected	Isolation – Infected	-2.943	0.014*

Supplementary Table 4 | Statistical differences for mortality between the different treatments presented in Fig 2a. Mixed effects Cox proportional hazards regression model: Random factor: Colony: Variance: 0.45, Std. Dev. 0.66. Likelihood ratio test of model vs intercept only model: X^2_3= 24.59, P<0.001. Post-hoc analysis with least square means with Holm-Bonferroni correction for the 4 groups.

State 1	State 2	*t* ratio	*P*
Nest – Sterile – 2h	Nest – Infected – 2h	0.061	1
Nest – Sterile – 2h	Isolation – Sterile – 2h	0.043	1
Nest – Sterile – 2h	Isolation – Infected – 2h	0.056	1
Nest – Sterile – 2h	Nest – Sterile – 11h	0.039	1
Nest – Sterile – 2h	Nest – Infected – 11h	0.024	1
Nest – Sterile – 2h	Isolation – Sterile – 11h	0.017	1
Nest – Sterile – 2h	Isolation – Infected – 11h	-4.776	<0.001***
Nest – Infected – 2h	Isolation – Sterile – 2h	-0.018	1
Nest – Infected – 2h	Isolation – Infected – 2h	-0.005	1
Nest – Infected – 2h	Nest – Sterile – 11h	-0.022	1
Nest – Infected – 2h	Nest – Infected – 11h	-0.037	1
Nest – Infected – 2h	Isolation – Sterile – 11h	-0.044	1
Nest – Infected – 2h	Isolation – Infected – 11h	-4.837	<0.001***
Isolation – Sterile – 2h	Isolation – Infected – 2h	0.013	1
Isolation – Sterile – 2h	Nest – Sterile – 11h	-0.004	1
Isolation – Sterile – 2h	Nest – Infected – 11h	-0.019	1
Isolation – Sterile – 2h	Isolation – Sterile – 11h	-0.026	1
Isolation – Sterile – 2h	Isolation – Infected – 11h	-4.819	<0.001***
Isolation – Infected – 2h	Nest – Sterile – 11h	-0.017	1
Isolation – Infected – 2h	Nest – Infected – 11h	-0.032	1
Isolation – Infected – 2h	Isolation – Sterile – 11h	-0.039	1
Isolation – Infected – 2h	Isolation – Infected – 11h	-4.832	<0.001***
Nest – Sterile – 11h	Nest – Infected – 11h	-0.015	1
Nest – Sterile – 11h	Isolation – Sterile – 11h	-0.023	1
Nest – Sterile – 11h	Isolation – Infected – 11h	-4.816	<0.001***
Nest – Infected – 11h	Isolation – Sterile – 11h	-0.007	1
Nest – Infected – 11h	Isolation – Infected – 11h	-4.800	<0.001***
Isolation – Sterile – 11h	Isolation – Infected – 11h	-4.793	<0.001***

Supplementary Table 5 | Statistical differences in bacterial load (*P. aeruginosa*) between the treatments presented in Fig 2b. Linear mixed effect model (Random Factor: Colony: Variance=$3.5*10^{-13}$, Std. Dev.=$5.8*10^{-7}$; Residual: Variance=50590, Std. Dev.=224.9; Likelihood ratio test of model vs intercept only model: X^2_9= 36.3, P<0.001), *n*=6 per group. Post-hoc analysis with least square means with Holm-Bonferroni correction for the 8 groups.

Mixed effects Cox Model

Experiment	Exp(coef)	*Z*	*P*
Isolation – clotted – Infected	9.07	3.13	<0.001***
Isolation – open – Sterile	1.32	0.46	0.65
Isolation – open – Infected	9.70	3.82	<0.001***
Nest – clotted – Sterile	0.30	-1.05	0.29
Nest – clotted – Infected	7.09	3.02	0.003**
Nest – open – Sterile	0.29	-1.07	0.28
Nest – open – Infected	1.02	0.02	0.98

Least square means (post-hoc analysis)

Experiment 1	Experiment 2	*Z*	*P*
Isolation – clotted – Sterile	Isolation – clotted – Infected	-3.387	0.015*
Isolation – clotted – Sterile	Isolation – open – Sterile	-0.455	1
Isolation – clotted – Sterile	Isolation – open – Infected	-3.818	0.003**
Isolation – clotted – Sterile	Nest – clotted – Sterile	1.050	1
Isolation – clotted – Sterile	Nest – clotted – Infected	-3.016	0.039*
Isolation – clotted – Sterile	Nest – open – Sterile	1.075	1
Isolation – clotted – Sterile	Nest – open – Infected	-0.020	1
Isolation – clotted – Infected	Isolation – open – Sterile	5.540	<0.001***
Isolation – clotted – Infected	Isolation – open – Infected	-0.210	1
Isolation – clotted – Infected	Nest – clotted – Sterile	3.271	0.019*
Isolation – clotted – Infected	Nest – clotted – Infected	0.603	1
Isolation – clotted – Infected	Nest – open – Sterile	3.298	0.019*
Isolation – clotted – Infected	Nest – open – Infected	3.364	0.015*
Isolation – open – Sterile	Isolation – open – Infected	-8.780	<0.001***
Isolation – open – Sterile	Nest – clotted – Sterile	1.464	1
Isolation – open – Sterile	Nest – clotted – Infected	-4.870	<0.001***
Isolation – open – Sterile	Nest – open – Sterile	1.493	1
Isolation – open – Sterile	Nest – open – Infected	0.429	1
Isolation – open – Infected	Nest – clotted – Sterile	3.447	0.012*
Isolation – open – Infected	Nest – clotted – Infected	0.983	1
Isolation – open – Infected	Nest – open – Sterile	3.476	0.012*
Isolation – open – Infected	Nest – open – Infected	3.793	0.004**
Nest – clotted – Sterile	Nest – clotted – Infected	-3.038	0.038*
Nest – clotted – Sterile	Nest – open – Sterile	0.021	1
Nest – clotted – Sterile	Nest – open – Infected	-1.064	1
Nest – clotted – Infected	Nest – open – Sterile	3.065	0.037*
Nest – clotted – Infected	Nest – open – Infected	2.992	0.039*
Nest – open – Sterile	Nest – open – Infected	-1.089	1

Supplementary Table 6 | Statistical differences for mortality between the different treatments presented in Supplementary Fig. 5. Mixed effects Cox proportional hazards regression model: Random factor: Colony: Variance: 0.00008, Std. Dev. 0.0089. Likelihood ratio test of model vs intercept only model: X^2_7= 119.9, *P*<0.001. Post-hoc analysis with least square means with Holm-Bonferroni correction for the 8 groups (28 tests). Nest: focal ant kept in sub-colony; Isolation: focal ant kept alone; clotted: MG clotted with acrylic color both in the focal ant and nestmates; open: MG was kept open both in the focal ant and nestmates; Sterile: wound of focal ant was exposed to PBS; Infected: wound of focal ant was exposed to 0.05 OD of *P. aeruginosa*.

Group 1	Group 2	R^2	*F*	*P*
Infected – 0h	Infected – 2h	0.13	2.42	0.05*
Infected – 0h	Infected – 11h	0.19	3.82	0.008**
Infected – 0h	Sterile – 0h	0.13	0.13	0.96
Infected – 0h	Sterile – 2h	0.11	1.95	0.09
Infected – 0h	Sterile – 11h	0.06	0.96	0.34
Infected – 2h	Infected – 11h	0.063	1.48	0.13
Infected – 2h	Sterile – 0h	0.17	3.26	0.005**
Infected – 2h	Sterile – 2h	0.008	0.17	0.87
Infected – 2h	Sterile – 11h	0.13	3.40	0.03*
Infected – 11h	Sterile – 0h	0.23	4.90	0.001***
Infected – 11h	Sterile – 2h	0.07	1.56	0.16
Infected – 11h	Sterile – 11h	0.19	5.4	0.007**
Sterile – 0h	Sterile – 2h	0.15	2.74	0.03*
Sterile – 0h	Sterile – 11h	0.03	0.53	0.45
Sterile – 2h	Sterile – 11h	0.14	3.54	0.04*

Supplementary Table 7 | Statistical differences in CHC-profile composition between the treatments. Permutational multivariate analysis of variance using Bray-Curtis dissimilarity matrices (ADONIS: formula: CHCdistancematrix ~ Treatment&Time*Sociality with Colony as random factor). Treatment&Time: Df=5, R^2=0.18, F=2.32, P=0.009; Sociality: Df=1, R^2=0.012, F=0.77, P=0.47; Treatment&Time*Sociality: Df=3, R^2=0.04, F=0.86, P=0.54; n=12 per group, except for timepoint 0: n=6. Detailed results for the Post-hoc analysis with a pairwise ADONIS with colony as random factor and correction for multiple comparisons (Holm Bonferroni) across the 6 groups are shown in the table.

Compound	Compound type	Ret. Index	Sterile 0h *n*=6	Sterile 2h *n*=12	Sterile 11h *n*=12	Infected 0h *n*=6	Infected 2h *n*=12	Infected 11h *n*=12
C20	Alkane	2000	0.0±0.1	0.1±0.1	0.1±0.1	0.0±0.0	0.0±0.0	0.1±0.0
C21	Alkane	2100	1.0±0.5	1.0±0.4	1.6±0.8	1.1±0.8	0.9±0.4	0.9±0.3
10-MeC21	Methyl	2140	0.6±0.1	0.5±0.2	0.5±0.2	0.4±0.1	0.4±0.2	0.5±0.2
3-MeC21	Methyl	2173	0.1±0.1	0.1±0.1	0.1±0.1	0.0±0.0	0.0±0.1	0.1±0.1
C22	Alkane	2200	0.4±0.4	0.3±0.1	0.4±0.1	0.4±0.6	0.3±0.1	0.4±0.2
C23en_1	Alkene	2273	1.6±1.4	1.7±0.9	1.3±0.7	1.7±1.8	1.4±0.5	1.5±0.8
C23en_2	Alkene	2280	0.0±0.0	0.0±0.0	0.0±0.0	0.0±0.0	0.0±0.0	0.0±0.1
C23	Alkane	2300	9.2±3.0	6.8±1.3	7.3±1.4	9.5±2.8	6.8±1.4	6.5±1.1
11-MeC23	Methyl	2335	1.2±0.6	1.3±0.2	1.2±0.4	1.2±0.7	1.1±0.3	1.2±0.3
5-MeC23	Methyl	2353	0.2±0.2	0.2±0.1	0.2±0.1	0.1±0.1	0.2±0.1	0.2±0.1
3-MeC23	Methyl	2372	0.5±0.5	0.2±0.3	0.2±0.3	0.1±0.1	0.0±0.0	0.4±0.1
C24	Alkane	2400	1.3±0.1	1.1±0.2	1.4±0.2	1.1±0.1	1.2±0.2	1.2±0.2
3,7-DiMeC23	Dimethyl	2409	0.0±0.0	0.0±0.0	0.1±0.2	0.0±0.0	0.0±0.1	0.2±0.1
C25en_1	Alkene	2471	0.5±0.4	0.6±0.4	0.5±0.3	0.5±0.7	0.5±0.2	0.7±0.4
C25en_2	Alkene	2478	0.0±0.0	0.1±0.1	0.1±0.1	0.0±0.0	0.0±0.1	0.1±0.1
C25	Alkane	2500	29.8±7.9	22.0±4.5	24.4±11.0	23.6±12.1	22.6±3.6	19.7±5.0
11-;13-MeC25	Methyl	2531	0.2±0.1	0.2±0.1	0.3±0.1	0.2±0.1	0.2±0.0	0.3±0.0
3-MeC25	Methyl	2572	0.3±0.2	0.2±0.0	0.2±0.1	0.1±0.1	0.2±0.1	0.2±0.1
C26	Alkane	2600	0.4±0.2	0.7±0.2	0.7±0.2	0.5±0.1	0.7±0.1	0.7±0.2
13-;11-;9-MeC26	Methyl	2614	0.0±0.0	0.0±0.0	0.0±0.0	0.0±0.0	0.0±0.0	0.1±0.1
C27en	Alkene	2676	1.2±0.4	1.7±0.4	1.4±0.3	1.4±1.0	1.7±0.4	1.8±0.3
C27	Alkane	2700	4.3±1.5	3.5±0.9	4.8±0.7	3.7±1.9	3.9±1.2	3.9±1.0
13-;11-MeC27	Methyl	2732	0.1±0.1	0.1±0.2	0.2±0.2	0.0±0.0	0.1±0.2	0.1±0.2
C29en_1	Alkene	2870	0.0±0.0	0.0±0.0	0.0±0.0	0.0±0.0	0.0±0.0	0.1±0.1
C29en_2	Alkene	2878	1.3±0.7	1.5±0.4	1.5±0.8	1.4±0.4	1.5±0.4	1.7±0.7
C29	Alkane	2900	0.8±0.2	1.0±0.3	1.1±0.2	0.9±0.2	1.0±0.2	1.2±0.3
15-;13-;11-MeC29	Methyl	2933	0.0±0.0	0.0±0.0	0.0±0.1	0.0±0.0	0.0±0.0	0.2±0.2
C30en	Alkene	2980	0.0±0.0	0.2±0.2	0.1±0.1	0.0±0.0	0.2±0.1	0.2±0.1
C31dien	Alkadiene	3050	0.6±0.2	1.0±0.3	0.7±0.5	0.5±0.3	0.8±0.3	1.2±0.5
C31en_1	Alkene	3070	0.4±0.5	0.7±0.2	0.6±0.4	0.3±0.4	0.8±0.3	0.9±0.3
C31en_2	Alkene	3084	19.4±3.8	20.8±1.0	18.5±2.2	19.9±4.9	21.0±0.9	19.8±1.1
C31	Alkane	3100	0.0±0.0	0.0±0.0	0.3±0.4	0.2±0.3	0.5±0.2	0.6±0.0
15-;13-;11-MeC31	Methyl	3130	0.0±0.0	0.1±0.2	0.1±0.1	0.0±0.0	0.1±0.1	0.2±0.2
C32dien	Alkadiene	3148	0.1±0.2	0.3±0.3	0.3±0.2	0.0±0.0	0.4±0.2	0.5±0.3
C33trien	Alkatriene	3232	0.0±0.0	0.1±0.0	0.0±0.0	0.0±0.0	0.1±0.1	0.1±0.0
C33dien_1	Alkadiene	3247	23.9±2.4	28.8±1.8	24.6±2.7	23.9±6.4	27.9±2.5	27.6±2.5
C33dien_2	Alkadiene	3274	0.0±0.0	0.0±0.0	0.0±0.0	0.0±0.0	0.1±0.1	0.4±0.5
C35trien	Alkatriene	3449	0.0±0.0	0.0±0.0	0.0±0.0	0.0±0.0	0.1±0.1	0.2±0.1
C35dien	Alkadiene	3472	0.0±0.0	0.0±0.0	0.0±0.0	0.0±0.0	0.0±0.0	0.1±0.1

Supplementary Table 8 | Chemical composition of the cuticular hydrocarbon profiles. Median relative percentages with median absolute deviation of all cuticular hydrocarbons between infected and sterile ants across the three timepoints (0h, 2h, 11h).

Group 1	Group 2	*P* adj.
Sterile – 0h	Sterile – 2h	0.01*
Sterile – 0h	Sterile – 11h	0.92
Sterile – 0h	Infected – 0h	0.99
Sterile – 0h	Infected – 2h	0.02*
Sterile – 0h	Infected – 11h	0.002**
Sterile – 2h	Sterile – 11h	0.04*
Sterile – 2h	Infected – 0h	0.07
Sterile – 2h	Infected – 2h	1
Sterile – 2h	Infected – 11h	0.98
Sterile – 11h	Infected – 0h	1
Sterile – 11h	Infected – 2h	0.049*
Sterile – 11h	Infected – 11h	0.005**
Infected – 0h	Infected – 2h	0.08
Infected – 0h	Infected – 11h	0.01*
Infected – 2h	Infected – 11h	0.96

Supplementary Table 9 | Statistical differences for alkadienes between the treatments presented in Supplementary Fig. 6. AOV model (alkanes: Df=5, Sum Sq=713.6, F=2.99, P=0.02 Residual: Df=53, Sum Sq=2522; alkenes: Df=5, Sum Sq=124.6, F=2.46, P=0.04; Residual: Df=53, Sum Sq=536; alkadienes: Df=5, Sum Sq=384.7, F=6.82, P<0.001 Residual: Df=53, Sum Sq=598; methyl-branched-alkanes: Df=5, Sum Sq=4.13, F=0.56, P=0.73 Residual: Df=53, Sum Sq=78) n=12 per group, except for timepoint zero were n=6 for sterile and infected ants. Post-hoc analysis with two-sided Tukey Honest Significant differences test. Even though the AOV was significant for alkanes and alkenes, the posthoc analysis with Holm-Bonferroni corrections for multiple testing across the 6 groups did not result in any pairwise significances, the detailed posthoc results shown in the table are thus only given for the alkadienes.

Gene IDs	Log2FoldChange	*P* adj.	Time	Putative Function	Biological Process (GO)
gene_22863	1.00068922	<0.001	2h	phosphatidylinositol 4-kinase beta	lipid biosynthetic process
gene_30894	2.36850382	<0.001	2h	glucose dehydrogenase	glucose metabolic process
gene_02329	-1.037557432	0.003	11h	endocuticle structural glycoprotein SgAbd-4	structural constituent of cuticle
gene_38881	-3.401621515	0.029	11h	sodium-coupled monocarboxylate transporter 1-like	short-chain fatty acid import
gene_08375	-1.532425715	<0.001	11h	lipid storage droplets surface-binding protein 2-like	regulation of lipid storage
gene_08376	2.55138413	0.048	11h	lipid storage droplets surface-binding protein 1	regulation of lipid storage
gene_31650	-2.305854998	<0.001	11h	palmitoyltransferase	acyltransferase activity
gene_34515	-1.801145986	0.003	11h	very long-chain-fatty-acid--CoA ligase bubblegum	long-chain fatty acid-CoA elongase activity
gene_40960	3.473641627	0.018	11h	elongation of very long chain fatty acids protein 1-like	fatty acid elongation
gene_14544	-4.357452189	0.002	11h	Cuticle protein 6	structural constituent of cuticle
gene_02681	1.051188424	<0.001	11h	phospholipase A2-like	lipid metabolic process
gene_14361	-1.56417983	0.045	11h	pancreatic triacylglycerol lipase	lipid metabolic process
gene_23311	1.428011778	0.027	11h	microsomal triglyceride transfer protein	lipid metabolic process
gene_29857	1.734871622	0.030	11h	diacylglycerol kinase eta	lipid metabolic process
gene_39381	-2.135617551	0.039	11h	alkaline ceramidase	lipid metabolic process
gene_18230	-0.613998454	0.004	11h	sterol O-acyltransferase	fatty-acyl-CoA binding
gene_39586	-0.944340779	0.012	11h	UDP-xylose and UDP-N-acetylglucosamine transporter-like	carbohydrate transport
gene_38486	2.738654673	<0.001	11h	sorbitol dehydrogenase-like	carbohydrate metabolism
gene_18615	-1.748361395	<0.001	11h	hydroxymethylglutaryl-CoA synthase 1	acetyl-CoA metabolic process
gene_38395	-1.037557432	0.045	11h	acid phosphatase	phosphoric ester hydrolase activity

Supplementary Table 10 | List of genes putatively implicated in CHC production and lipid metabolism being differentially expressed between sterile and infected ants presented in Fig. 5. Positive Log2FoldChange values correspond to genes up-regulated in infected ants. Significant differences were calculated using a two-sided Wald test and corrected for multiple testing using the Benjamini and Hochberg method

Gene IDs	Log2FoldChange	*P* adj.	Time	Putative Function	Biological Process (GO)
gene_02403	2.35332709	0.001	2h	tyrosine kinase receptor Cad96Ca-like	positive regulation of wound healing
gene_07900	1.07975465	0.009	2h	peptidoglycan-recognition protein SC2-like PGRP-LB	positive regulation of Toll signaling pathway
gene_12381	5.27341349	<0.001	2h	Pirk	negative regulation of peptidoglycan recognition protein signaling pathway
gene_14574	2.45236598	0.001	2h	Abaecin	hemolymph coagulation
gene_25734	5.05363939	<0.001	2h	Hymenoptaecin	innate immune response
gene_25736	4.00166659	<0.001	2h	Hymenoptaecin	innate immune response
gene_39135	1.3685947	0.003	2h	serine protease inhibitor 88Ea-like	negative regulation of innate immune response (Toll)
gene_01714	2.68170259	0.006	11h	PDGF- and VEGF-related factor 3	hemocyte migration
gene_07900	2.55345461	<0.001	11h	peptidoglycan-recognition protein SC2-like PGRP-LB	positive regulation of Toll signaling pathway
gene_11103	0.85188693	0.006	11h	fas-associated death domain protein (Fadd)	positive regulation of innate immune response
gene_12381	5.63944847	<0.001	11h	Pirk	negative regulation of peptidoglycan recognition protein signaling pathway
gene_14574	4.429955	<0.001	11h	Abaecin	hemolymph coagulation
gene_17612	1.56736442	<0.001	11h	phenoloxidase-activating factor 2 (PPAF2)	innate immune response
gene_25734	7.21006972	<0.001	11h	Hymenoptaecin	innate immune response
gene_25736	6.31954344	<0.001	11h	Hymenoptaecin	innate immune response
gene_25792	0.65380865	0.021	11h	Autophagy-related protein 16-1 (Atg 16)	positive regulation of autophagy
gene_29419	-1.8089618	0.003	11h	protein croquemort-like	immune response-regulating cell surface receptor signaling pathway involved in phagocytosis
gene_30388	0.40053527	0.009	11h	coronin-1C-A (coro)	defence response to fungus
gene_34607	1.95877062	<0.001	11h	Cytokine receptor	receptor signaling pathway via JAK-STAT
gene_37992	0.77372933	<0.001	11h	2-oxoglutarate-dependent dioxygenase htyE (2OGDDs)	biosynthesis of the beta-lactam antibiotics
gene_39135	2.15522495	<0.001	11h	serine protease inhibitor 88Ea-like	negative regulation of innate immune response (Toll)
gene_39979	2.55922106	<0.001	11h	serine protease easter (ea)	positive regulation of Toll signaling pathway
gene_41380	1.49200932	<0.001	11h	serine protease gd	positive regulation of Toll signaling pathway
gene_41384	2.08260184	<0.001	11h	serine protease gd	positive regulation of Toll signaling pathway

Supplementary Table 11 | Immune system related genes differentially expressed between sterile and infected ants presented in Fig. 5. Positive Log2FoldChange values correspond to genes up-regulated in infected ants. Significant differences were calculated using a two-sided Wald test and corrected for multiple testing using the Benjamini and Hochberg method

D. mel ortholog	Protein IDs	Function	Orthology Depth	% MG Content	<20 kDa	Implications for wound healing from orthologs
-	gene_35433	Unknown	None	13.239	X	
CG15203	gene_35399	Unknown	Arthropoda, poss. slime mold	5.943	X	Toxin-like, O_Venom
Hml	gene_35649	chymotrypsin inhibitor	Endopterygota	5.568	X	O_Hemocyte, O_Venom
-	gene_41829	Unknown	Hymenoptera	4.237	X	Toxin-like, O_Venom
straw	gene_12257	laccase	Pterygota	3.192		O_Melanization
yellow-d	gene_33077	MRJP	Hymenoptera	2.919		O_Antimicriobial
straw	gene_12258	laccase	Pterygota	1.935		O_Melanization
Gba1b	gene_20433	glucosylceramidase	Bilateria	1.077		
yellow-b	gene_33078	MRJP	Hymenoptera	0.797		O_Antimicrobial
CG34034	gene_25950	omega-conotoxin-like	Insecta, poss. bacteria, fungi	0.751	X	Toxin-like, O_Venom
-	gene_00214	Unknown	Formicidae	0.646	X	Toxin-like, O_Venom
-	gene_13637	Unknown	Formicidae, possibly sawfly	0.586	X	
CG6426	gene_37453	lysozyme	Neoptera	0.431	X	O_Antimicrobial
-	gene_16798	kielin/chordin-like	Endopterygota	0.244		
-	gene_25492	Unknown	Formicidae	0.236	X	Toxin-like, O_Venom
eater	gene_29492	VWDE / eater / fibrilin	Neoptera	0.181		O_Hemocyte
-	gene_25504	Odorant binding protein, GP9-like	Aculeata	0.168	X	
-	gene_25951	omega-conotoxin-like	Insecta, poss. bacteria, fungi	0.147	X	Toxin-like, O_Venom
-	gene_00213	Unknown	Formicidae	0.128	X	O_Venom
crok	gene_28725	Unknown/quiver	Neoptera	0.124	X	O_Venom
-	gene_24061	Unknown	Hymenoptera, poss. bacteria	0.107	X	O_Venom
CG42259	gene_35648	chymotrypsin inhibitor	Invertebrates, fungi, viruses	0.070	X	O_Wound_response, Toxin-like, O_Venom
CG8369	gene_09378	kazal-type proteinase inhibitor / vasotab	Neoptera	0.051	X	O_Vasodilator
CG9917	gene_38892	Interferon-related developmental regulator	Pterygota	0.043		
-	gene_20297	Myrosinase-1-like	Endopterygota	0.036		
Nep2	gene_06446	Neprilysin / membrane metallo-endopeptidase	Pterygota	0.034		
CG30197	gene_25140	waprin-Thr1	Neoptera	0.027	X	Toxin-like, O_Venom, O_Antimicrobial
CG30197	gene_25139	waprin-Phi1	Pteroygota	0.023		Toxin-like, O_Venom, O_Antimicrobial
-	gene_08904	CREG1-like	Pancrustacea	0.021		
CG15140	gene_38752	Prisilkin/trithorax/pro-resilin	Endopterygota	0.021		
-	gene_16797	kielin/chordin-like	Endopterygota	0.020		
hgo	gene_00180	homogentisate 1,2-dioxygenase	Bilateria	0.019		O_Melanization
CG6414	gene_06365	Venom carboxylesterase-6	Neoptera	0.014		
-	gene_00215	Unknown	Formicidae, possibly bacteria	0.011	X	O_Venom
l(1)G0289	gene_07578	plexin domain-containing protein	Pterygota	0.011		
CAH2	gene_03491	carbonic anhydrase	Pterygota	0.008		
mgl	gene_13013	sortilin-related receptor	Pterygota	0.007		
Alp4	gene_33376	alkaline phosphatase	Hexapoda	0.006		
Fkbp14	gene_11011	FK506 Binding protein, TOR related	Pancrustacea, fungi	0.006	X	
Atpα	gene_07025	sodium/potassium-transporting ATPase	Bilateria	0.004		
Nrx-1	gene_10345	Multiple EGF-like domains / crumbs	Arthropoda	0.003	X	Toxin-like

Supplementary Table 12 | Proteins from the metapleural gland. Forty-one proteins had a significantly greater abundance in the metapleural gland atrium than in the hemolymph (see Supplementary Fig. 7b). Abbreviations: X: Low molecular weight (<20kDa); O_: function found in an orthologous protein; *Drosophila melanogaster* orthologs are indicated where there was sufficient similarity. Proteins are sorted by their abundance in the metapleural gland atrium. Function orthology depth (common sequences) and implications were based on protein BLAST hits using the experimental clustered nr database[37].

Compound	Ret. Index	Relative abundance	Function / Chemical Group
Unidentified_1	842	0.22±0.26	NA
Furanmethanol	860	0.31±0.15	Alcohol
Similar to 2-Propenamide	900	0.17±0.21	Amide
Similar to 2-Propenamide	905	0.17±0.18	Amide
2(5H)-Furanone	918	0.31±0.07	
3-Methyl-2(5H)-furanone	982	0.44±0.18	
Phenol	993	0.21±0.10	**Acid**
Hexanoic acid	1016	0.22±0.12	**Acid**
Unidentified_2	1033	0.05±0.05	NA
Unidentified_3	1049	0.13±0.07	NA
3-Ethyl-2,5-dimethyl-pyrazine	1083	0.57±0.51	**Alkaloid**
Unidentified_4	1086	0.30±0.09	NA
Unidentified_5	1088	0.27±0.28	NA
Unidentified_6	1091	0.39±0.29	NA
Hexanamide	1129	0.06±0.05	Amide
Unidentified_7	1143	0.40±0.50	NA
2,3-Dihydro-3,5-dihydoxy-6-methyl-4H-pyran-4-one	1153	0.15±0.15	
Glutarimide	1160	0.13±0.05	Amide
Unidentified_8	1174	0.09±0.13	NA
Catechol	1215	0.18±0.18	**Antimicrobial**
Unidentified_9	1263	0.20±0.13	NA
Nonanoic acid	1288	0.42±0.23	**Acid**
Indole	1299	0.93±0.31	Amine
Unidentified_10	1303	0.39±0.28	
Unidentified_11	1310	0.30±0.31	NA
4-Methyl-1,2-benzenediol	1318	0.20±0.26	NA
2,6-Dimethoxyphenol	1359	0.41±0.38	
Unidentified_12	1364	0.36±0.23	NA
Unidentified_13	1368	0.30±0.32	NA
3-Methyl-indole	1391	0.63±0.17	**Alkaloid**
5-Oxo-L-proline methyl ester	1396	0.56±0.77	**Alkaloid**
Alkaloid_1	1410	0.80±0.35	**Alkaloid**
Alkaloid_2	1414	0.34±0.34	**Alkaloid**
Similar to Gougerotin	1446	0.12±0.12	**Antibiotic**
Caprolactone derivative	1466	0.48±0.39	
Isoxacol derivative	1470	0.17±0.10	**Antimicrobial component**
Unidentified_14	1473	0.23±0.20	NA
Alkaloid_3	1475	0.38±0.20	**Alkaloid**
Piperidine derivative_1	1479	0.63±0.14	**Alkaloid**
Piperidine derivative_2	1484	0.58±0.33	**Alkaloid**
Piperidine derivative_3	1489	0.24±0.02	**Alkaloid**
Unidentified_15	1511	0.12±0.12	NA
Unidentified_16	1515	0.16±0.23	NA
Pyrimidin derivative	1520	0.11±0.12	
Alkaloid_4	1549	0.28±0.18	**Alkaloid**
Alkaloid_5	1553	0.34±0.00	**Alkaloid**
Dodecanoic acid	1581	2.12±1.28	**Acid**
Unidentified_17	1587	0.40±0.24	NA
Alkaloid_6	1617	0.42±0.14	**Alkaloid**
Alkaloid_7	1626	0.68±0.17	**Alkaloid**
Unidentified_18	1630	0.25±0.31	NA
12-Hydroxydecanoic acid	1666	0.86±0.79	**Acid**
10-Hydroxydecanoic acid	1671	2.06±1.91	**Acid**
Alkaloid_8	1673	0.23±0.22	**Alkaloid**
Deoxyspergualin derivative	1679	0.31±0.30	**Antibiotic**

Unidentified_19	1682	0.31±0.12	NA
Unidentified_20	1689	0.28±0.26	NA
Alkaloid_9	1704	0.16±0.05	**Alkaloid**
Alkaloid_10	1712	0.33±0.18	**Alkaloid**
Similar to 3-Methyl-1,4-diazabicyclo[4.3.0]nonan-2,5-dione	1732	3.44±1.12	
Alkaloid_11	1739	0.69±0.39	**Alkaloid**
Alkaloid_12	1747	0.29±0.06	**Alkaloid**
Similar to 3-Methyl-1,4-diazabicyclo[4.3.0]nonan-2,5-dione	1758	2.02±0.46	
Alkaloid_13	1770	0.54±0.38	**Alkaloid**
Alkaloid_14	1780	3.42±1.30	**Alkaloid**
Alkaloid_15	1783	1.60±0.72	**Alkaloid**
Alkaloid_16	1785	0.95±0.86	**Alkaloid**
Alkaloid_17	1796	0.43±0.42	**Alkaloid**
Alkaloid_18	1806	0.23±0.05	**Alkaloid**
Alkaloid_19	1810	0.36±0.31	**Alkaloid**
Alkaloid_20	1817	0.40±0.09	**Alkaloid**
Alkaloid_21	1828	0.47±0.53	**Alkaloid**
Actinomycin C2 derivative_1	1830	0.74±0.83	**Antibiotic**
Pentadecanoic acid_1	1845	0.18±0.08	**Acid**
Alkaloid_22	1861	0.60±0.37	**Alkaloid**
Alkaloid_23	1864	0.22±0.19	**Alkaloid**
Alkaloid_24	1870	0.29±0.30	**Alkaloid**
Pentadecanoic acid_2	1875	0.50±0.17	**Acid**
Alkaloid_25	1894	0.62±0.30	**Alkaloid**
Alkaloid_26	1898	0.13±0.13	**Alkaloid**
Hydrocarbon	1930	0.60±0.56	CHC
Alkaloid_27	1932	0.43±0.22	**Alkaloid**
Alkaloid_28	1946	1.79±0.75	**Alkaloid**
Alkaloid_29	1949	0.62±0.54	**Alkaloid**
Hexadecenoic acid_1	1953	0.62±0.69	**Acid**
Hexadecanoic acid_2	1986	17.65±0.80	**Acid**
Unidentified_21	1999	0.12±0.12	NA
Heptadecenoic acid_1	2050	0.32±0.06	Acid
Alkaloid_30	2054	0.59±0.16	**Alkaloid**
Heptadecanoic acid_2	2068	0.27±0.04	**Acid**
Octadecanol	2085	0.23±0.17	Alcohol
Octadecadienoic acid methyl ester	2096	0.67±0.15	Ester
Unidentified_22	2113	0.30±0.05	NA
Octadecanoic acid methyl ester	2129	0.41±0.05	Ester
Octadecenoic acid_1	2152	21.44±9.09	**Acid**
Octadecanoic acid_2	2176	7.80±1.04	**Acid**
Actinomycin C2 derivative_2	2237	0.15±0.13	**Antibiotic**
Fatty acid methyl ester	2261	0.28±0.03	Ester
Eicosanol	2286	0.24±0.02	Alcohol
2-(8Z)-8-Heptadecen-1-yl-4,5-dihydro-oxazole	2319	0.17±0.15	
Fatty acid ester_1	2358	0.68±0.13	Ester
Octadecenamide	2364	0.95±0.20	Amide
Octadecanamide	2389	0.25±0.04	Amide
Unidentified_23	2414	0.18±0.07	NA
N,N-Dimethyl-octadecenamide	2434	0.23±0.06	Amide
2-(Dimethylamino)ethyl-octadecadienoate	2456	0.34±0.08	
2-(Dimethylamino)ethyl-octadecenoate	2461	0.65±0.12	
Hexadecanoic acid, 2-hydroxy-1-(hydroxymethyl)ethyl ester	2509	0.20±0.09	Ester
Fatty acid ester_2	2543	0.16±0.07	Ester
Fatty acid ester_3	2565	0.22±0.18	Ester
Dodecanoic acid, hexadecyl ester	2954	0.21±0.06	Ester
Fatty acid ester_4	3126	0.21±0.07	Ester

Supplementary Table 13 | Chemical compounds in the metapleural gland. Table includes information on compounds found in the MG samples (Supplementary Fig. 8). Chemical groups written in bold are known to have antimicrobial effects. Six MG were pooled per sample run in the GC-MS-TD (*n*=3).
