# Supplementary material for: Targeted treatment of injured nestmates with antimicrobial compounds in an ant society
Source: Nat Commun. 2023 Dec 29;14:8446. doi: 10.1038/s41467-023-43885-w (PMC10756881; doi:10.1038/s41467-023-43885-w)
Supplement: Supplementary file 3 — Description of Additional Supplementary Files [file 41467_2023_43885_MOESM3_ESM.pdf]

File Name: Supplementary Data 1
Description: Supplementary Figures 1 to 8 and Supplementary Tables 1 to 13

File Name: Supplementary Data 2
Description: Workflow for hierarchical generalized additive model for woundcare behaviour. Detailed reproducible workflow for the statistical analyses and graphical illustration of Fig. 3b and 3c. File Name: Supplementary Data 3 Description: Zip-file with R-code for all graphical illustrations and raw Data and code for Microbiome analyses

File Name: Supplementary Movie 1
Description: Example of wound care performed with metapleural gland secretions collected from the gland of the individual providing care. The infected ant is marked in white. We first observe wound care by the nursing ant, followed by the collection of metapleural gland secretions using the forelegs to reach the gland and mouth and finally application of metapleural gland secretions on the wound.

File Name: Supplementary Movie 2
Description: Example of wound care performed with metapleural gland secretions collected from the gland of the injured individual. The infected ant is marked in white and red. We first observe wound care by the nursing ant, followed by the collection of metapleural gland secretions of the injured individual using its mouthparts to reach the gland and finally application of metapleural gland secretions on the wound.
