# Supplementary material for: Targeted treatment of injured nestmates with antimicrobial compounds in an ant society
Source: Nat Commun. 2023 Dec 29;14:8446. doi: 10.1038/s41467-023-43885-w (PMC10756881; doi:10.1038/s41467-023-43885-w)
Supplement: Supplementary file 8 — Reporting Summary [file 41467_2023_43885_MOESM8_ESM.pdf]

nature portfolio

Corresponding author(s): Dr. Erik T. Frank

Last updated by author(s): Nov 15, 2023

# Reporting Summary

Nature Portfolio wishes to improve the reproducibility of the work that we publish. This form provides structure for consistency and transparency in reporting. For further information on Nature Portfolio policies, see our Editorial Policies and the Editorial Policy Checklist.

## Statistics

For all statistical analyses, confirm that the following items are present in the figure legend, table legend, main text, or Methods section.

| n/a | Confirmed | |
|---|---|---|
| ☐ | ☒ | The exact sample size (*n*) for each experimental group/condition, given as a discrete number and unit of measurement |
| ☐ | ☒ | A statement on whether measurements were taken from distinct samples or whether the same sample was measured repeatedly |
| ☐ | ☒ | The statistical test(s) used AND whether they are one- or two-sided<br>*Only common tests should be described solely by name; describe more complex techniques in the Methods section.* |
| ☐ | ☒ | A description of all covariates tested |
| ☐ | ☒ | A description of any assumptions or corrections, such as tests of normality and adjustment for multiple comparisons |
| ☐ | ☒ | A full description of the statistical parameters including central tendency (e.g. means) or other basic estimates (e.g. regression coefficient) AND variation (e.g. standard deviation) or associated estimates of uncertainty (e.g. confidence intervals) |
| ☐ | ☒ | For null hypothesis testing, the test statistic (e.g. *F*, *t*, *r*) with confidence intervals, effect sizes, degrees of freedom and *P* value noted<br>*Give P values as exact values whenever suitable.* |
| ☒ | ☐ | For Bayesian analysis, information on the choice of priors and Markov chain Monte Carlo settings |
| ☐ | ☒ | For hierarchical and complex designs, identification of the appropriate level for tests and full reporting of outcomes |
| ☒ | ☐ | Estimates of effect sizes (e.g. Cohen's *d*, Pearson's *r*), indicating how they were calculated |

*Our web collection on statistics for biologists contains articles on many of the points above.*

## Software and code

Policy information about availability of computer code

| | |
|---|---|
| Data collection | Data collection was done manually in the field. |
| Data analysis | All analyses were conducted in R (v4.1.0) using RStudio (v1.4.1717) and the code is provided in the data repository. R-packages used during this study include: ggplot2 (v3.3.5), vegan (v2.6-4), reshape2 (v1.4.4), MASS (v7.3-60), randomForest (v4.7-1.1), adegenet (v2.1.10), dendextend (v1.17.1), colorspace (v2.1-0), RColorBrewer (v1.1-3), gplots (v3.1.3), ggbeeswarm (v0.7.2), magrittr (v2.0.3), ggpubr (v0.6.0), lme4 (v1.1-35), nlme (v3.1-163), emmeans (v1.8.9), survival (v3.5-7), splines (v4.3.2), survminer (v0.4.9), coxme (v2.2-18.1). |

For manuscripts utilizing custom algorithms or software that are central to the research but not yet described in published literature, software must be made available to editors and reviewers. We strongly encourage code deposition in a community repository (e.g. GitHub). See the Nature Portfolio guidelines for submitting code & software for further information.

## Data

Policy information about availability of data

All manuscripts must include a data availability statement. This statement should provide the following information, where applicable:

- Accession codes, unique identifiers, or web links for publicly available datasets
- A description of any restrictions on data availability
- For clinical datasets or third party data, please ensure that the statement adheres to our policy

The raw amplicon-sequence data generated in this study have been deposited at the Sequence Read Archive (SRA) under accession code PRJNA826317 [https://www.ncbi.nlm.nih.gov/sra/?term=PRJNA826317]. The sequence reads data generated in this study have been deposited in NCBI Sequence Read Archive (SRA)

under accession code PRJNA823913 [https://www.ncbi.nlm.nih.gov/sra?term=PRJNA823913]. The proteomics data generated in this study have been deposited at the ProteomeXchange Consortium via the PRIDE partner repository under accession code PXD033003 [https://proteomecentral.proteomexchange.org/cgi/GetDataset?ID=PXD033003]. The CHC and MG data generated via GC-MS in this study have been deposited at the Dryad repository under the doi:10.5061/dryad.hqbzkh1j6 [https://datadryad.org/stash/share/gFLLhPMhmWW8HJ_JaKyTqg9Eq3QXqGS_EgxYEQvKKe8]. The Genome Assembly data are available under restricted access due to it being part of another Publication within the GAGA Project, access can be obtained by contacting the corresponding Author of the GAGA Project. Source data are provided as a Source Data file.

## Research involving human participants, their data, or biological material

Policy information about studies with human participants or human data. See also policy information about sex, gender (identity/presentation), and sexual orientation and race, ethnicity and racism.

| | |
|---|---|
| Reporting on sex and gender | NA |
| Reporting on race, ethnicity, or other socially relevant groupings | NA |
| Population characteristics | NA |
| Recruitment | NA |
| Ethics oversight | NA |

Note that full information on the approval of the study protocol must also be provided in the manuscript.

# Field-specific reporting

Please select the one below that is the best fit for your research. If you are not sure, read the appropriate sections before making your selection.

☐ Life sciences ☐ Behavioural & social sciences ☒ Ecological, evolutionary & environmental sciences

For a reference copy of the document with all sections, see nature.com/documents/nr-reporting-summary-flat.pdf

# Ecological, evolutionary & environmental sciences study design

All studies must disclose on these points even when the disclosure is negative.

| | |
|---|---|
| Study description | This study includes experiments in behaviour, microbiology, chemical ecology, proteomics, genomics and microCTscans. In all experiments the main treatment factor was comparing infected wounds to sterile wounds either at fixed times (0, 2 and 11 hours) or over time (0 to 24 hours). When comparisons were conducted between isolated ants or individuals kept in colonies this was included as a second treatment factor (isolation or colony). Whenever applicable colony of origin was included as a random factor to the statistical analyses. |
| Research sample | All experiments were conducted on female workers of the species Megaponera analis in the Comoé National Park (North Eastern Côte d'Ivoire). The species is found throughout sub-Saharan Africa from 25°S to 12°N. |
| Sampling strategy | Sample sizes where chosen to allow in a cost and time-efficient way to determine significant differences between treatments. By only conducting experiments under controlled experimental conditions with only one variable being changed across treatments we further felt confident in reducing sample sizes ranging from 6 to 15 across experiments. A sample size often used in experiments of this nature (see Frank et al. 2017 and Frank et al. 2018). |
| Data collection | All field data was collected by Erik Frank. Behavioural data was collected by Andrei Dascalu from video recordings. Microbiological data was extracted from samples by Lucie Kesner and Joanito Liberti (samples were collected by Erik Frank). Gene expression data was extracted from samples by Quentin Helleu (samples were collected by Erik Frank). micro CT scans were conducted by Evan Economo from samples send by Erik Frank. |
| Timing and spatial scale | Experiments, observations, and sample collections in the Comoé National Park were carried out from April to June 2018, February, April to June and September to October 2019, April 2020 and October 2022. The break in data collection between 2020 and 2022 was caused by the COVID-19 Pandemic. |
| Data exclusions | No data was excluded from the analyses. |
| Reproducibility | Survival experiments were repeated with a positive (infected wound) and negative (sterile wound) during each experiment conducted between 2018-2022. The results were reproducible every time (6 times in total). |
| Randomization | All workers collected from the colonies for the experiments were done so in a random manner from the female foraging population. |
| Blinding | For behavioural and survival experiments a blinded approach was conducted in which the observer did not know the treatment (infected or sterile wound) of the focal individuals. Due to the standardized protocol in the microbiological, genomics, chemical and proteomic analyses we considered a blinded approach unnecessary in these experiments. |

Did the study involve field work? ☒ Yes ☐ No

## Field work, collection and transport

| | |
|---|---|
| Field conditions | Field work was carried out at the Comoé National Park Research Station in North Eastern Côte d'Ivoire. The climate is tropical with temperatures over the year ranging from 20.6-32.2°C and distinct dry and rainy seasons. The wet season lasts over 6 months distributed in two periods with a longer season from March to June and a shorter season from September to October. The annual precipitation ranges from 1500 – 2200 mm with the highest precipitation in June (180mm) and October (120mm). |
| Location | The study was conducted in the Comoé National Park, located in the North-eastern region of Côte d'Ivoire at the Comoé National Park Research Station (8°46'N 3°47'W). |
| Access & import/export | All samples were collected in the surrounding of the research station (walking distance). The research conducted in this study complies with all relevant ethical regulations and was approved by the park management of Office Ivoirien des Parcs et Réserves (OIPR) in Côte d'Ivoire as part of the bilateral research agreement between Germany (represented by the University of Würzburg) and Côte d'Ivoire (represented by OIPR). The ants collected for this study are part of the bilateral research agreement under research permit number N°018 / MINEDD / OIPR / DZ. |
| Disturbance | Ant colonies collected from the field were returned to their original location at the end of the experiments to minimize disturbance. |

# Reporting for specific materials, systems and methods

We require information from authors about some types of materials, experimental systems and methods used in many studies. Here, indicate whether each material, system or method listed is relevant to your study. If you are not sure if a list item applies to your research, read the appropriate section before selecting a response.

## Materials & experimental systems

| n/a | Involved in the study | |
|---|---|---|
| ☒ | ☐ | Antibodies |
| ☒ | ☐ | Eukaryotic cell lines |
| ☒ | ☐ | Palaeontology and archaeology |
| ☐ | ☒ | Animals and other organisms |
| ☒ | ☐ | Clinical data |
| ☒ | ☐ | Dual use research of concern |
| ☒ | ☐ | Plants |

## Methods

| n/a | Involved in the study | |
|---|---|---|
| ☒ | ☐ | ChIP-seq |
| ☒ | ☐ | Flow cytometry |
| ☒ | ☐ | MRI-based neuroimaging |

## Animals and other research organisms

Policy information about studies involving animals; ARRIVE guidelines recommended for reporting animal research, and Sex and Gender in Research

| | |
|---|---|
| Laboratory animals | The study did not involve laboratory animals |
| Wild animals | Colonies of Megaponera analis were collected in full in the field (digging out the colony). Since colonies of M. analis reproduce through colony fission, age cannot be determined and colony size always represents a "mature" stage. All colonies were of a normal size (between 1000 and 2000 individuals). At the end of the experiments (2-3 months) the colonies were placed again in the same location from where they were extracted. The colonies were excavated at walking distance from the field laboratory and transported by foot in plastic containers shielded from the sun and kept under humid conditions. The strain for the ants is not available. |
| Reporting on sex | All ant workers are female, therefore we did not report on sex. |
| Field-collected samples | Eleven colonies were excavated and placed in artificial nests in the field stations laboratory (colony size 1083±258 ants, n=11), including queen and brood. Nests (30x20x10 cm) were made of PVC and connected to a 1x1m feeding arena. The ground and nest were covered with soil from the surrounding area (up to a height of 2 cm). Macrotermes bellicosus termites were collected from the surrounding area by using pots filled with dry grass and placed in the feeding arena. These termites were found by scouts and triggered raiding behavior. The laboratory windows were kept open to maintain a natural humidity, temperature, and day-night cycle (light regime). |
| Ethics oversight | No ethical approval or guidance is required for ants. |

Note that full information on the approval of the study protocol must also be provided in the manuscript.
